# Has the Time Come to Stratify and Score SUDEP Risk to Inform People With Epilepsy of Their Changes in Safety?

**DOI:** 10.3389/fneur.2018.00281

**Published:** 2018-04-27

**Authors:** Rohit Shankar, Craig Newman, Alistair Gales, Brendan N. McLean, Jane Hanna, Samantha Ashby, Matthew C. Walker, Josemir W. Sander

**Affiliations:** ^1^Cornwall Partnership NHS Foundation Trust, Truro, United Kingdom; ^2^Exeter Medical School, Knowledge Spa, Royal Cornwall Hospital, Truro, United Kingdom; ^3^Plymouth University Peninsula Schools of Medicine and Dentistry, Plymouth, United Kingdom; ^4^Wairau Hospital, Blenheim, New Zealand; ^5^Royal Cornwall Hospital, Truro, United Kingdom; ^6^SUDEP Action, Wantage, United Kingdom; ^7^NIHR University College London, Hospitals Biomedical Research Centre, UCL Institute of Neurology, London, United Kingdom; ^8^Chalfont Centre for Epilepsy, Buckinghamshire, United Kingdom; ^9^Stichting Epilepsie Instellingen Nederland (SEIN), Heemstede, Netherlands

**Keywords:** risk factors, communication, classify, risk assessment, SUDEP

## Abstract

Recent publication of the American Academy of Neurology SUDEP guidance highlighted the importance to American clinicians of making people with epilepsy aware of SUDEP risk. It is the first guideline to do this in the United States. It follows precedent set out in the UK by National Institute of Clinical Excellence in 2004. While a significant achievement, the lack of clarity of how to deliver this guidance in an enduring and person-centered manner, raises concerns on how its long-term effectiveness in risk mitigation. Shared decision-making with an emphasis on delivering person-centered communication to foster self-management strategies is increasingly recognized as the ideal model of patient–clinician communication in chronic diseases such as epilepsy. The tension between delivering evidence-based risk information, yet, tailoring it to the individual is complex. It needs to incorporate the potential for change not only in seizure factors but also other health and social factors. Safety advice needs to be dynamic and situation sensitive as opposed to a “one off” discussion. As a significant minority of people with epilepsy have drug-resistant seizures, the importance of keeping the advice contextual at different intervals of the person’s life cannot be overstated as many of them are managed in primary care. We present some exploratory work, which identifies the need to improve communication at a primary care level and to review risks regularly. Regular reviews using a structured risk factor checklist as a screening tool could identify, sooner, people who’s health issues are worsening and justify referrals to specialists.

Living is a risky business. Risk is the chance that an activity or action could lead to harm. Currently, there is no such option as zero risk. People will generally modify behavior and change lifestyle if they feel that there is a person-centered advantage or benefit. To bring about such a change, people need to know and comprehensively understand specific risks. Health risks are conveyed to individuals by clinicians in a myriad of ways of variable quality and effectiveness. As there are no specific “rules,” a host of clinician and individual factors play a role in person-centered communication (1). The lack of a clear structure to capture systemically this quality of communication could influence outcomes. The current level of evidence and the availability of a structure to deliver the information needed for individuals to be able to understand and be more aware of their distinct risk underpin the strength of individual risk avoidance.

New guidance from The American Academy of Neurology (AAN) recognizes the importance of communicating the risk of Sudden Unexpected Death in Epilepsy (SUDEP) to people with epilepsy (2). It is a welcome document, the first of its kind for American clinicians, which was critically developed and which provides clarity on the current level of evidence of the various risk factors highlighted to date in the literature. It also establishes the importance of a discussion of these risks with individuals mirroring the position taken by the UK’s National Institute of Clinical Excellence (NICE) (3) guidance for epilepsy since 2004. While this is a major step forward, similar to NICE, the AAN guidance lacks elaboration on how to deliver person-centered risk assessment. It stops short of providing guidance or support to empower clinicians in having this critical conversation in clinic. Another concern with such guidance is that the discussion of SUDEP risk is expected to occur once at the time or near the time of diagnosis. There seems to be a lack of recognition of the importance to keep updating risk assessment and feedback based on the course an individual’s epilepsy takes and how this may change. It has been shown in chronic conditions, epilepsy in particular that risk can change over time and is heavily influenced by varying life factors (4). People with epilepsy, often to their detriment, are rarely aware of changing risk. Thus, what can start as “low” risk can over time switch to a “higher” risk without the individual or the care giver or even the clinician being fully aware.

It is important that risk assessments in epilepsy are person-centered, contextual, and focused on the “here and now,” and reviewed at regular intervals to ensure that there is a full picture of individual risk status. It is worth noting that most people with epilepsy, especially in countries where there are public health systems, as in the UK, are not usually supported by specialist epilepsy services but mainly by primary care where knowledge of up-to-date epilepsy and associated risk issues may be lacking and vary significantly. This can also be case when neurologists who are not epilepsy specialists (5) are involved.

The extent of the problem is highlighted by the fact that in 2013 in the UK around 1,200 people died due to epilepsy, which was roughly the same number who died from asthma in the same year (6). This is despite the number of people with asthma being 10 times larger (6). The data from National Statistics suggested that up to 60% of the epilepsy deaths could have been avoidable while only a quarter of asthma deaths were identified as preventible (6). This suggests that there is significant room for improvement in the way risk identification and management of people with epilepsy happen in the community.

In primary care (the General Practice system), epilepsy remains a common and regular presentation with Public Health expenditure in the UK for neurological conditions, second only to stroke (7, 8). Around 600 people with epilepsy in the UK die of SUDEP each year (7, 9). It is likely that these statistics underrepresent the true number of epilepsy deaths each year in the UK.

In 2004, an incentivizing scheme for the provision of quality care and to standardize improvement in the delivery of primary care was introduced in England called Quality Outcome Framework (QOF). The focus was to encourage primary care to manage common chronic conditions and enable the implementation of preventative strategies (10). Epilepsy had four QOF outcome indicators, one of which included an annual monitoring of people aged 18 and over on drug treatment. Epilepsy drug treatment monitoring was withdrawn from the scheme in 2014, raising concerns as there are now less opportunities to review individual risk changes as a result of the abandonment of the annual reviews. No clear evidence has, however, emerged as yet to the impact of this (11).

The SUDEP and Seizure Safety Checklist (“Checklist”)^1^ is a free, practical, evidence-based tool available in the UK for regular clinical use (12, 13). Its aim is to help person-centered communication to empower individuals and families with epilepsy to take shared responsibility with clinicians to make meaningful changes to improve their seizure risk outcomes. It also enables clinicians to identify change and compare with baseline in a structured manner. Description of the Checklist is provided in Appendix 1.

The Checklist has 19 modifiable and non-modifiable factors, providing an outline for clinician’s discussions with individuals, which can be repeated annually or when a person with unstable epilepsy is reviewed urgently or routinely. Clinicians in secondary care have found the tool practical and time efficient (10 min), but it has not yet been systematically used in primary care though anecdotal feedback is that it is used significantly by clinicians working in primary care or out in the community.

The concept has been tested as a telehealth project for a year in a single site large primary care practice in mid-Cornwall having 16,000 people registered to it to risk assess “high risk” individuals with epilepsy in the catchment (14). “High risk” was defined as over 10 years of treatment-resistant seizures but “stable” in the community. The telehealth team called on a three monthly basis and ran the Checklist with the registered users. All results were communicated back to the GP. Of the 46 people with epilepsy in the practice who received the telehealth screening, 17 were referred for several interventions during the year that would not have happened without the on-going screening. However, a problem of this study was that it identified “high risk” based on a single factor of 10 years treatment resistance and not on a holistic risk issue. Thus, another study was set up to look at all registered people with epilepsy in a different primary care practice.

The setting was a medium sized primary care facility covering a mixed urban and large rural area in SW England covering around 12,000 people. A database identifying all people with epilepsy in the practice with a baseline risk score was proposed using the Checklist factors. The purpose was to allow for rapid re-valuation of risk status during an annual review or consultation to help identify seizure risk change using the Checklist to help recognize, categorize, and stratify seizure risk. Person-centered advice could then be provided based on the changed findings. Using the facilities, standard digital clinical management system, EMIS Web (EmisHealth, Leeds, UK), a search was created to identify anyone with epilepsy, epileptiform conditions, or seizures. Each risk factor of the Checklist was then identified and relating codes were then automatically searched in the database. This was done for each individual, thus allowing individual risk score analysis as well as analysis of the population.

A total of 107 target individuals were identified. All 19 risk factors were applied. The mean score was 4.1. The range was from 1 to 9 though only two people scored 9 and three had scores of 8. There were no data coded or any documented evidence for four of the risk factors (nocturnal surveillance, pregnancy, prone sleeping position, and nocturnal seizure presence) in any individual.

Undertaking this evaluation resulted in each of the individuals at the primary care facility undergoing a baseline screen and s risk factors scoring. It has also identified common risk factors and individual modifiable factors. The perceived lack of awareness for risk among primary care clinicians and the lack of clinical codes to identify the presence of major established risk factors is of concern. Given the overall high standards followed in this facility, it could be expected that similar shortcomings would be seen in other practices leading to concerns of the gap in knowledge and awareness.

These data were presented to the facility clinical practitioners (physicians and nurses) and the realization that some risk factors may have been missed in certain individuals’ stimulated discussion. This was highlighted by the discussion around the risk factor of nocturnal seizures where no coding data were present. Facility practitioners acknowledged that it was not a practice to ask of nocturnal events during reviews. As this is a modifiable high risk factor, this warrants full incorporation into records as it may help reduce risk in certain patient-groups. Indeed, as a result of these findings, the annual epilepsy review structure of the facility was changed to include the Checklist risk factors.

The strength of the Checklist was its ability to motivate risk considerations of SUDEP with people with epilepsy and their families. All 107 identified when reviewed and given feedback felt the conversation was useful again confirming the importance of person-centered discussion of risk. It showcased the importance of sharing risk knowledge in giving ownership to people with epilepsy and their carers. This supports findings of another recent study where the structured use of the Checklist in specialist epilepsy clinics led to the reduction of risk scores. It has helped possibly reduce the burden of SUDEP in the long term. It should be stressed that for most risk factors, it is not clear if a modification of these factors changes the actual SUDEP risk and this is an area which needs further exploring (2).

This exercise has also allowed positive discussions and learning among physicians, nurses, and individuals within the facility about SUDEP and risk factors and has acted as a catalyst to hopefully improve care, monitoring and outcomes in the longer term. It also highlights the value of risk identification and coding in epilepsy community care, through the use of the clinical Checklist. The value of education and empowerment is intuitive in all areas of clinical risk reduction and is particularly relevant in epilepsy.

This intervention was well received and is easily translatable to most primary care settings, so the following recommendations would be reasonable:

1. People with epilepsy should have an annual seizure safety risk assessment at primary care.
2. An earlier interim assessment needs to be triggered if any person with epilepsy presents with: decline in seizure control, alteration to AEDs or relevant medications, change in comorbidities in particular use of alcohol or other substances or with psychiatric issues.

It is appreciated that the postulated move to stratify risk with the current level of evidence might be a controversial one. It could be argued that such an attempt could confound and cause fright. An example was the 1995 “pill scare” when third generation Oral contraceptive pills were proposed to double venous thromboembolism risk compared with older alternatives; however, this was later established not to be the case (15). In the interim, there was a noted increase in unintended pregnancies and increased rates of abortions as a result of the “pill scare.” This example highlights an extreme situation especially where new data or research have been used. For chronic conditions, given the diversity and cumulative effects of numerous factors over a lifetime, a perfect risk assessment is unlikely to be delivered. In the case of epilepsy, people will still continue to prematurely die if steps are not put in place to reduce their risks. Those bereaved by the condition and those who have supported interventions are united in their determination to stop any unnecessarily deaths. In such situations, evidence-based pragmatism with a focus on improving individual wellbeing and safety is the way forward. Risk stratification, while not ideal in current day practice, could be the lowest common acceptable denominator to structure current evidence into small bite-size packages of information to improve knowledge of clinicians, measure and map risk, and empower individuals as part of a holistic approach to epilepsy risk management as highlighted by this small study.

It is important to note that creating such baseline scores and risk stratification for known risk factors is still only a first step in improving awareness among clinicians and people in such settings. Enabling them to then have risk discussions using tools such as the Checklist, and work together to reduce these risks where possible must follow to help tackle premature mortality. There have been other attempts to provide collective risk factors toward SUDEP most notably the SUDEP-7 Inventory (16). The SUDEP-7 Inventory similar to the Checklist has undergone a range of testing. Commonalities with the Checklist include the use of similar background literature to evidence the risk factors (17). The differences are in the focus of the inventory, target group, and its purpose. The SUDEP-7 risk factor items are primarily concentrated on seizures and are correlated to electro-physiological variations (18). Its principal role is to provide a screening inventory focused on biomarkers (18). This is different from the ambitions of the Checklist, which looks to communicate person-centered risk to individuals with a view to empowering them to make necessary adaptations in their day-to-day life (19). As the two tools work differently, there could be a case made to use them symbiotically. This concept would require further testing.

The manner of discussing SUDEP is not without challenges. These challenges are diverse and include personal, professional, cultural, institutional, and resource issues (20, 21). Personal and professional beliefs of whether it makes a difference to discuss are still an ongoing debate (20, 21). Cultural attitudes may also play a role (21). Availability of trained physicians itself is a concern in many parts of the world. Where there are services available, often there is no time or space to have such sensitive conversations as about SUDEP. Thus, while SUDEP discussion and continued risk mitigation may be a “step too far” in many areas, it would still be the practice to aspire to.

## Ethics Statement

We confirm that we have read the journal’s position on issues involved in ethical publication and affirm that this report is consistent with those guidelines. No ethical approval was needed as this was a service improvement project.

## Author Contributions

All authors contributed to the concept, development of the paper, editing, and finalizing of it. RS wrote the initial draft with CN. AG collected data. BM, LS, and MW gave supervision and oversight.

## Conflict of Interest Statement

RS, JH, and SA are the main stakeholders of the “SUDEP and Seizure Safety Checklist.” RS, JH, SA, BM, and CN are developers and key stakeholders of EpSMon. MW and JS are members of the SUDEP and Seizure Safety Checklist review panel. JS has received departmental research support from GSK, Eisai, and UCB Pharma and has been consulted by and received fees for lectures from Bial, Eisai, and UCB Pharma outside the submitted work. MW has received departmental research support from Vitaflo and has been consulted by and received fees for lectures from GSK, Pfizer, Eisai, and UCB outside the submitted work. RS has received institutional and research support and personal fees from LivaNova, UCB, Eisai, Special Products, Bial, and Desitin outside the submitted work. BM has received research support and personal fees from Eisai, UCB, and Desitin outside the submitted work.

## Footnotes

^1^https://www.sudep.org/checklist (Accessed: October 28, 2017).

## Appendix 1

### The SUDEP and Seizure Safety Checklist (“Checklist”) and the EpSMon (22, 23) (As Described by SUDEP Action & Plymouth University)

The Checklist is a free, award-winning risk assessment tool for clinicians, which encompasses known modifiable and non-modifiable risk factors of SUDEP and associated concerns with a view to:

- Assist clinicians to open a positive discussion with people about epilepsy and risk assessment;
- Support a person-centered discussion of risk, focusing on whether known risk factors apply to a particular individual;
- Help clinicians educate people with epilepsy about their personal risk and possible lifestyle changes, which might reduce those risks;
- Promote the safety goal by identifying modifiable risk factors which may guide management;
- Create documentary evidence for clinicians on the impact of the treatment plan over time and demonstrate effective clinical governance while enhancing individual safety;
- Provide some assurance to bereaved families that every effort was made to reduce risk and prevent a fatality when a death occurs.

An example on how to administer is provided^1^; clinicians can also register for the tool, or find out more *via* www.sudep.org/checklist. The Checklist is managed by SUDEP Action (Secretariat and PPI Leads) and Cornwall Partnership NHS Foundation Trust (Clinical Leads).

The Checklist also underpins the content of a mobile app for people with epilepsy, EpSMon, which has been recognized as one of eight innovations selected for the current NHS Innovation Accelerator Programme.^2^ The App brings lifesaving information to the fingertips of adults with epilepsy, enabling them to monitor their own seizures and well-being between medical appointments. EpSMon also shows whether risk factors have improved or worsened enabling people with epilepsy to seek medical help sooner if required. It is free to download for iPhone and Android devices in the UK; further information can be found at www.epsmon.com. EpSMon is a partnership between Cornwall Partnership NHS Foundation Trust, SUDEP Action, Plymouth University, and Royal Cornwall Hospitals Trust. The Checklist and EpSMon are also part of the UK Epilepsy Commissioning Toolkit.^3^

The introduction of an app delivering education and risk assessment is innovative in respect to current practice, but easily perceived as an efficient route to providing best practice. The app’s ability to prompt timely assessments, assess current understanding, track engagement, deliver bespoke education, and recommend clinical support could be invaluable to the care for epilepsy in the community. Future identified developments will include medication adherence tools, the development of manualized approaches for services to implement the Checklist and EpSMon app alongside each other and will look to explore the potential of automatic flagging of at risk individuals to health teams. Use of it to reduce potential harm has been strongly supported by a recent Cochrane review on SUDEP prevention (24) and a National Institute of Health Research UK Systematic appraisal of emerging technologies for the diagnosis, treatment, and management of epilepsy (25).

^1^https://www.youtube.com/watch?v=Z9KHQvsapAc. ^2^https://www.youtube.com/watch?v=e3mECsSVgHI. ^3^http://www.epilepsytoolkit.org.uk/.
